# An Overlooked Hepcidin–Cadmium Connection

**DOI:** 10.3390/ijms232415483

**Published:** 2022-12-07

**Authors:** Dawid Płonka, Marta D. Wiśniewska, Manuel D. Peris-Díaz, Artur Krężel, Arkadiusz M. Bonna, Wojciech Bal

**Affiliations:** 1 Institute of Biochemistry and Biophysics, Polish Academy of Sciences, Pawińskiego 5a, 02-106 Warsaw, Poland; 2 Department of Chemical Biology, Faculty of Biotechnology, University of Wrocław, Joliot-Curie 14a, 50-383 Wrocław, Poland

**Keywords:** hepcidin, prohepcidin, cadmium, affinity constant, metallothionein, MT2A

## Abstract

Hepcidin (DTHFPICIFCCGCCHRSKCGMCCKT), an iron-regulatory hormone, is a 25-amino-acid peptide with four intramolecular disulfide bonds circulating in blood. Its hormonal activity is indirect and consists of marking ferroportin-1 (an iron exporter) for degradation. Hepcidin biosynthesis involves the N-terminally extended precursors prepro-hepcidin and pro-hepcidin, processed by peptidases to the final 25-peptide form. A sequence-specific formation of disulfide bonds and export of the oxidized peptide to the bloodstream follows. In this study we considered the fact that prior to export, reduced hepcidin may function as an octathiol ligand bearing some resemblance to the N-terminal part of the α-domain of metallothioneins. Consequently, we studied its ability to bind Zn(II) and Cd(II) ions using the original peptide and a model for prohepcidin extended N-terminally with a stretch of five arginine residues (5R-hepcidin). We found that both form equivalent mononuclear complexes with two Zn(II) or Cd(II) ions saturating all eight Cys residues. The average affinity at pH 7.4, determined from pH-metric spectroscopic titrations, is 10^10.1^ M^−1^ for Zn(II) ions; Cd(II) ions bind with affinities of 10^15.2^ M^−1^ and 10^14.1^ M^−1^. Using mass spectrometry and 5R-hepcidin we demonstrated that hepcidin can compete for Cd(II) ions with metallothionein-2, a cellular cadmium target. This study enabled us to conclude that hepcidin binds Zn(II) and Cd(II) sufficiently strongly to participate in zinc physiology and cadmium toxicity under intracellular conditions.

## 1. Introduction

Metals in organisms (both essential and non-essential) share similarities that make their regulating pathways overlap to some extent [1]. Thus, all elements of the metallome puzzle ought to be extensively investigated. One of them, hepcidin, is an iron-regulatory hormone. In its mature form it is a 25-amino-acid peptide with four intramolecular disulfide bonds that circulates in blood (DTHFPICIFCCGCCHRSKCGMCCKT). Its hormonal activity is indirect and consists of marking ferroportin-1 (an iron exporter) for degradation. This stops the iron efflux from cells [2].

Hepcidin itself does not bind iron but has two distinct metal-binding sites with a potentially high affinity for other metal ions. One of these sites is its N-terminal tripeptide sequence DTH, which belongs to the ATCUN (Cu- and Ni-binding site) family [3,4]. The mature hepcidin-25 indeed has a high affinity for Cu(II), and can also weakly bind Ni(II) and Zn(II), the latter two unlikely to have a biological meaning [3,5].

The other putative metal-binding site can be proposed based on the layout of thiol groups in the molecule. The eight cysteines in hepcidin are arranged in a similar manner to those in the N-terminal part of the α-domain of metallothioneins, proteins involved in metal homeostasis and detoxification [6,7] (see Scheme S1). However, for hepcidin to properly function as an iron hormone, only one of these cysteines is essential. The rest do not have an apparent function except to potentially increase the peptide’s stability in blood [8]. Nevertheless, all eight cysteines are evolutionarily conserved.

When hepcidin is circulating in blood, all the cysteines are arranged in disulfides within one molecule and with a specific pattern of these bonds [9]. However, before their release into the bloodstream, hepcidin and its immature forms prepro- and pro-hepcidin are biosynthesized, processed, and trafficked in the reducing, thiol-rich environment of the cell. The standard path for hepcidin maturation involves transfer to the endoplasmatic reticulum (ER) [10], where most probably the disulfides form [11]. Later it is cleaved by furin and secreted [12]. However, there are also reports stating that prohepcidin may be present in cytoplasm or even the nucleus [13,14]. Normally, hepcidin is fully oxidized and secreted between 1.5 and 6 h after exposure to inflammation [15], but there are reports that even fully oxidized peptides such as defensins are susceptible to thioredoxin disulfide cleavage [16]. Therefore, some degree of reversibility and disulfide exchange can happen. Thus, with the cysteines exposed hepcidin may be a molecular target for thiophilic metals such as cadmium.

Cadmium is a toxic metal targeting a number of organs. Its toxicity is pleiotropic and manifests throughout the body, but mainly in kidneys, liver, or lungs, dependent on the type of exposure [17]. Acute dietary cadmium poisoning damages the liver while chronic exposure eventually reaches and damages the kidneys [18]. The assumption is that it is excreted with metallothioneins and then filtered in nephrons, but it can be also transported with other low- and high-molecular-mass proteins [19].

It was reported that hepcidin is upregulated by cadmium exposure [20,21], although that observation was contested [22]. Interestingly, hepcidin-gene (*Hamp1*) silencing decreases Cd^2+^ toxicity in mice inner medullary collecting duct (mIMCD_3_) cells. Similarly, overexpression of *Hamp1* increases Cd^2+^ toxicity [23]. A full mechanism for this phenomenon is still unknown.

In addition to mature hepcidin, prohepcidin can also be detected in blood and urine, but unlike the properly processed peptide, it is not correlated with iron levels in blood.

The present paper explores the possibility of hepcidin binding Cd^2+^ ions on a molecular level to provide a background for continued studies of the hepcidin-cadmium link. Zn^2+^ binding was also explored, due to the intrinsic physiological zinc/cadmium link [7].

## 2. Results

**Figure 1:**
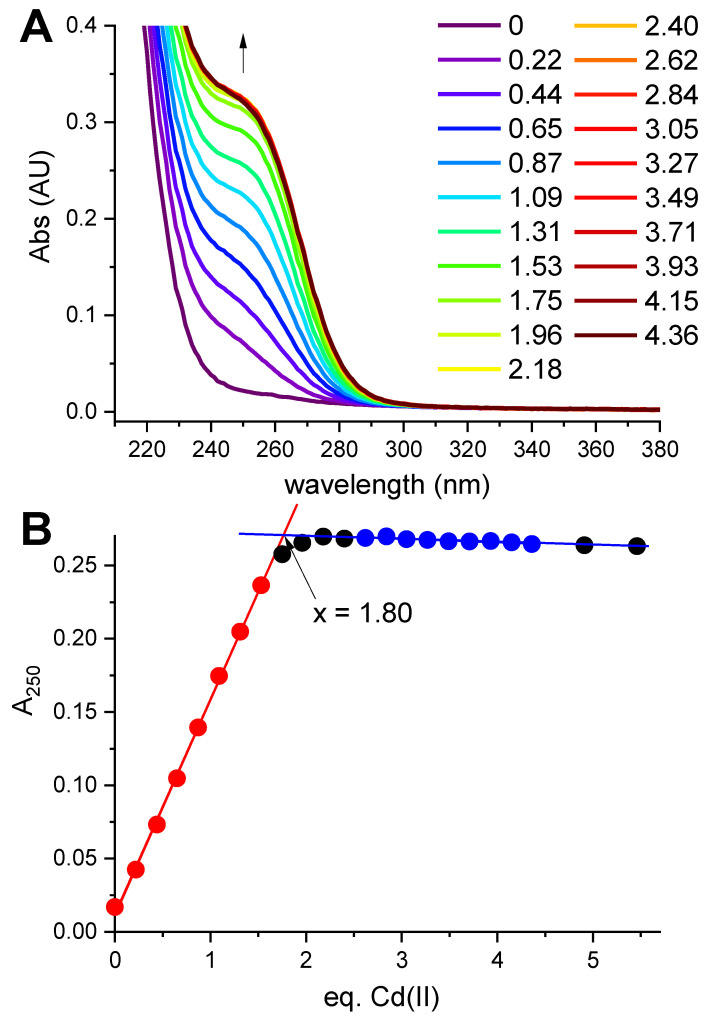
Titration of 12.57 μM hepcidin-25 with CdCl_2_ in the presence of 0.5 mM tris(2-carboxyethyl)phosphine (TCEP) at pH 5.0. (**A**). The spectra featuring the CT band at 250 nm formed upon the increasing Cd(II)/hepcidin-25 ratios (color coded in the plot); the arrow marks the direction of changes; (**B**). The titration curve at 250 nm. The red and blue segments of the curve were used to define the straight lines of the binding-site saturation. Their crossing at 1.80 Cd(II) equivalents determines the saturation concentration.

Hepcidin-25 was synthesized using standard Fmoc solid-phase synthesis, and its identity and the reduced state were confirmed using mass spectrometry (monoisotopic peak +3H^+^ of the peptide with free thiols was 932.707). In the first spectroscopic experiment reduced hepcidin-25 (12.57 μM dissolved in the presence of 0.5 mM TCEP at pH 5.0 to maintain the reduced character of all eight Cys residues) was titrated with a CdCl_2_ solution. A characteristic charge-transfer (CT) Cd(II)-thiolate band [24] emerged at 250 nm (Figure 1A).

The full saturation of this band with two Cd(II) equivalents is visible in Figure 1B. The value 1.8 in the plot, compared to equilibrium saturation 1.9 at pH 5.0 (see below), indicated a 5% deficit of hepcidin-25 and was used to correct its stock solution concentration in further experiments.

**Figure 2:**
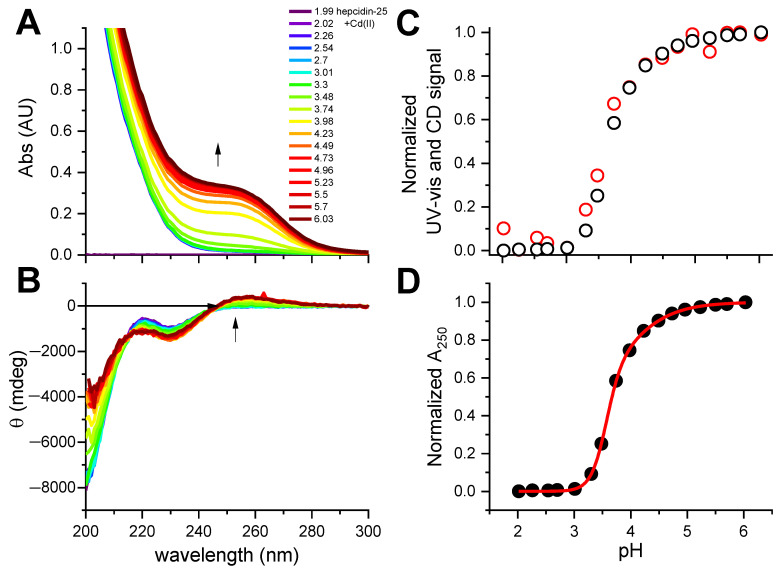
The pH-metric titration of 12.2 μM hepcidin-25 with 2 eq. of CdCl_2_ in the presence of 0.5 mM TCEP. (**A**). UV-vis spectra; (**B**). CD spectra, with the common color codes for pH in plot A; arrows mark the increase of the Cd(II)-S CT bands; (**C**). The pH dependence of CT bands at 250 nm derived from UV-vis (black dots) and CD (red dots) spectra, presented in the common relative scale; (**D**). Fitting of the two-sites binding model to the normalized UV-vis data at 250 nm.

This experiment established the 2:1 Cd(II)-to-hepcidin-25 stoichiometry. The linear increase of A_250_ indicated that the Cd(II) sites are spectroscopically equivalent. In the next experiment hepcidin-25 was thus mixed with two Cd(II) equivalents at pH 2.0 and titrated pH-metrically using a concentrated NaOH solution. This titration was monitored with UV-vis and CD spectroscopies (Figure 2A,B). Again, a distinct CT band appeared at 250 nm in UV-vis and CD spectra above pH 3. Figure 2C presents the titration curves generated from this titration, and Figure 2D provides the fitting of the two-sites binding model for the UV-vis data (less noisy than the qualitatively equivalent CD data). These titrations could not be extended beyond pH 6.0 due to peptide precipitation.

The fitting of the titration curve derived from the pH dependence of the Cd(II)-S CT band, presented in Figure 2D, yielded p*K*_exp_ values for the binding of two Cd(II) ions of 3.60 ± 0.01 and 3.88 ± 0.02. Assuming the average p*K* of the thiol group (p*K*_SH_) in Hepc-25 as 8.9, by analogy with metallothionein [25], and the structurally equivalent binding of both Cd(II) ions to four Cys residues each, one can tentatively extrapolate these p*K* values into log *K* values for absolute Cd(II) affinity constants as high as 21.2 and 20.1, and conditional constants at pH 7.4 as 15.2 and 14.1, according to Equations (1) and (2).

absolute log *K* = 4 × (p*K*_SH_ − p*K*_exp_) (1)

conditional log *K*_7.4_ = 4 × (7.4 − p*K*_exp_) (2)

**Figure 3:**
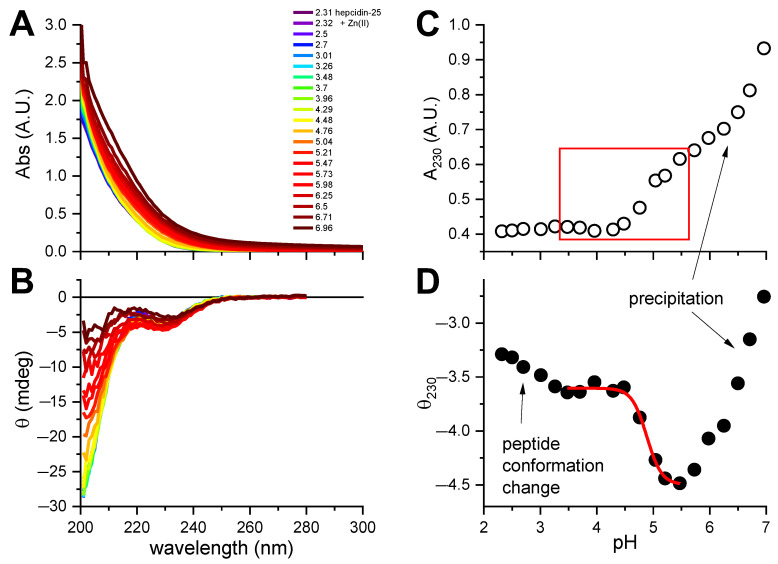
The pH-metric titration of 15.5 μM hepcidin-25 with 2 eq. of ZnCl_2_ in the presence of 0.5 mM TCEP. (**A**). UV-vis spectra; (**B**). CD spectra, with the common color codes for pH in plot A; (**C**). The pH dependence of A_230_ in the UV-vis spectra, showing the Zn(II) binding range (red box) and the loss of solution transparency due to peptide precipitation; (**D**). Fitting of the one-site binding model to the CD data at 230 nm. Arrows point to pH regions pertaining to comments in plots.

Unfortunately, we were not able to obtain p*K*_SH_ by spectroscopic titrations, because hepcidin-25 precipitated above pH 7. A similar pH-dependent experiment was carried out for hepcidin-25 with two equivalents of Zn(II) added (Figure 3). A CT band around 220 nm (Figure 3A) was hardly visible in the spectra due to its weakness, in accord with the previous data for tetrathiolate zinc fingers and metallothioneins [7,26] compounded by peptide precipitation above pH 5, clearly seen in Figure 3C. However, the change of ellipticity around 230 nm (Figure 3B) could be assigned to a combination of conformational effects in the peptide main chain and Zn(II) binding. The region of the titration curve tentatively assigned to be Zn(II)-dependent was used to fit the p*K* for Zn(II) binding to hepcidin-25, as presented in Figure 3D. The average log *K* value obtained from this titration, 4.88 ± 0.03, can be extrapolated into the log *K* value for absolute Zn(II) affinity constant of 16.1, and conditional constant at pH 7.4 of 10.1, according to Equations (1) and (2), respectively. This value should be treated as very tentative, because it is based on the assumption of Cys4 coordination. It is possible, but not certain, as hepcidin also contains two His residues which can participate in Zn(II) binding [27].

A more direct estimation of affinity constants for Cd(II) and Zn(II) complexes with hepcidin-25 could not be performed due to a very strong binding. Very poor solubility of the peptide near the neutral pH posed additional experimental problems. We therefore attempted several competition approaches. We first tried a stepwise competition experiment using Co(II) and Ni(II) ions, which bind to thiol peptides with weaker affinities. We successfully applied this strategy before for XPA and PARP zinc finger peptides [26,28,29]. Unfortunately, this method failed for hepcidin-25, because of a different, 3:1 stoichiometry (hence non-equivalent substitutions) for Co(II) and peptide precipitation for Ni(II). We also tried to use small competitors with varying Cd(II) affinities, EDTA, HEDTA, and EGTA, as described in [30]. Unfortunately, all three anionic chelators caused a partial precipitation of hepcicin-25, and no meaningful conclusions could be drawn.

In order to alleviate the problem of the solubility of hepcidin-25, its sequence was extended N-terminally by 5 arginines, which are the last five residues of prohepcidin. The new peptide (RRRRRDTHFPICIFCCGCCHRSKCGMCCKT) was labelled 5R-hepcidin. The arginine residues in prohepcidin are recognized by furin, the protease involved in hepcidin maturation [12]. Notably, prohepcidin has no additional binding sites for metal ions, and exists intracellularly. Therefore, any interaction of metals with hepcidin thiols should also be represented by 5R-hepcidin. The 5R addition indeed increased the peptide’s solubility at least fivefold and improved its detection by electrospray mass spectrometry (MS) in the positive-ions mode. Therefore, we attempted to determine the thiol p*K* values for this peptide. Unfortunately, the presence of two His residues obscured the spectral effects of Cys deprotonations up to pH 8, and the peptide precipitated at pH 9.6. This precluded the determination of thiol p*K* values (see Figure S1 in the SI). The only p*K* values obtained by the fitting of this curve could be assigned to the Asp side-chain carboxyl (4.18 ± 0.03) and to a His residue (6.66 ± 0.05). Other apparent values emerge from the overlap of His and Cys side-chain deprotonations and cannot be assigned reliably to either side-chain type.

**Figure 4:**
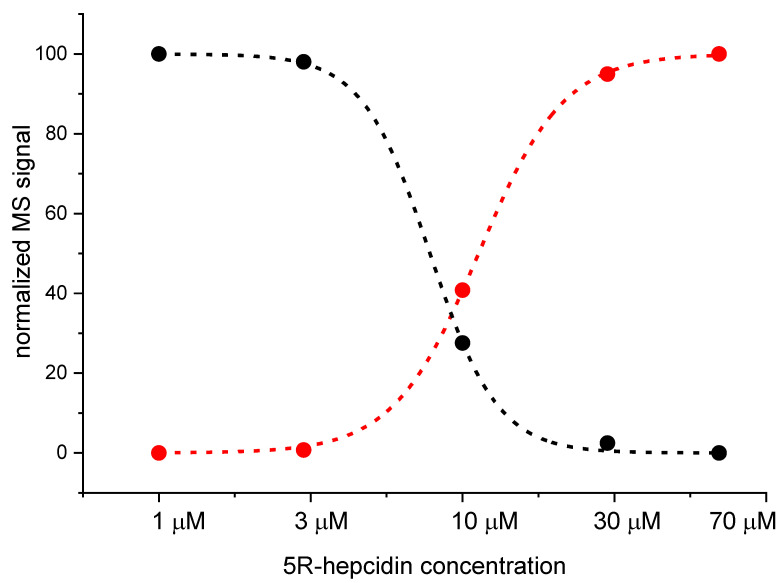
Integrated native MS signals of Cd_2_hepcidin-25 (black dots) and Cd_2_5R-hepcidin (red dots) obtained from the titration of 10 μM Cd_2_hepcidin-25 with 5R-hepcidin. The lines represent fits for the competition constant for the reaction Cd_2_hepcidin-25 + 5R-hepcidin = hepcidin-25 + Cd_2_5R-hepcidin. The MS spectra indicated only low amounts of other metallated species (see Figure S3).

As expected, 5R-hepcidin retained the ability to bind Cd(II) and Zn(II) ions demonstrated for hepcidin-25 (Figure S2). The apparent maximum metal-to-peptide ratio of 1.5 noted in this experiment is most likely due to partial peptide thiol oxidation during sample handling. This enabled us to perform a direct competition experiment between hepcidin-25 and 5R-hepcidin. Since both have essentially the same binding site, distinguishing it by spectroscopic methods would be impossible. Native mass spectrometry was thus employed to detect any differences. Cd_2_hepcidin-25 in ammonium acetate at pH = 7.4 was titrated with rising concentrations of 5R-hepcidin. The samples were injected on a Q-exactive UHMR mass spectrometer designed to preserve non-covalent bonds. One should always be wary of potential errors and differences in ionization, bonds not being carried over to gas phase or general lack of solvent during measurement [31]. However, in this case Cd(II) is not a fast-exchanging metal ion and the peptides were similar enough to consider this technique. This titration, illustrated in Figure 4 (see Figure S3 for the spectra), confirmed our assumption of the equivalence of both peptides with respect to Cd(II) binding, with the relative binding affinity of hepcidin-25/5R-hepcidin of 1.1 ± 0.3.

**Figure 5:**
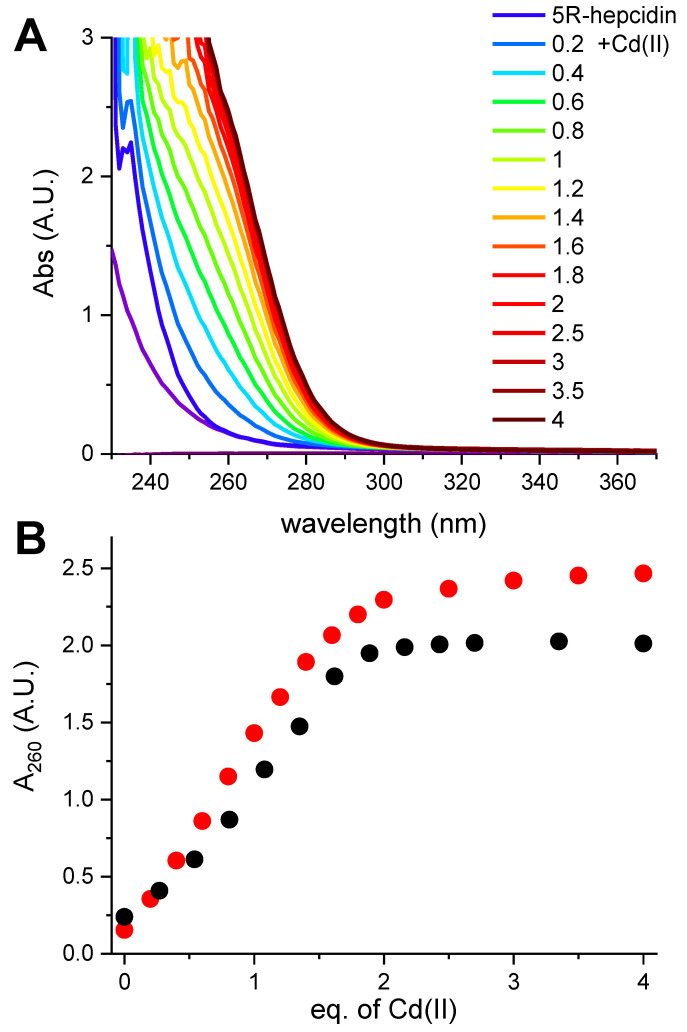
(**A**). 100 μM Zn_2_5R-hepcidin titrated with Cd(II) acetate in 100 mM HEPES pH 7.4. (**B**). The comparison of A_260_ values for Cd(II) titrations of 100 μM 5R-hepcidin (black dots, data from Figure S2) and Zn_2_5R-hepcidin (red dots).

**Figure 6:**
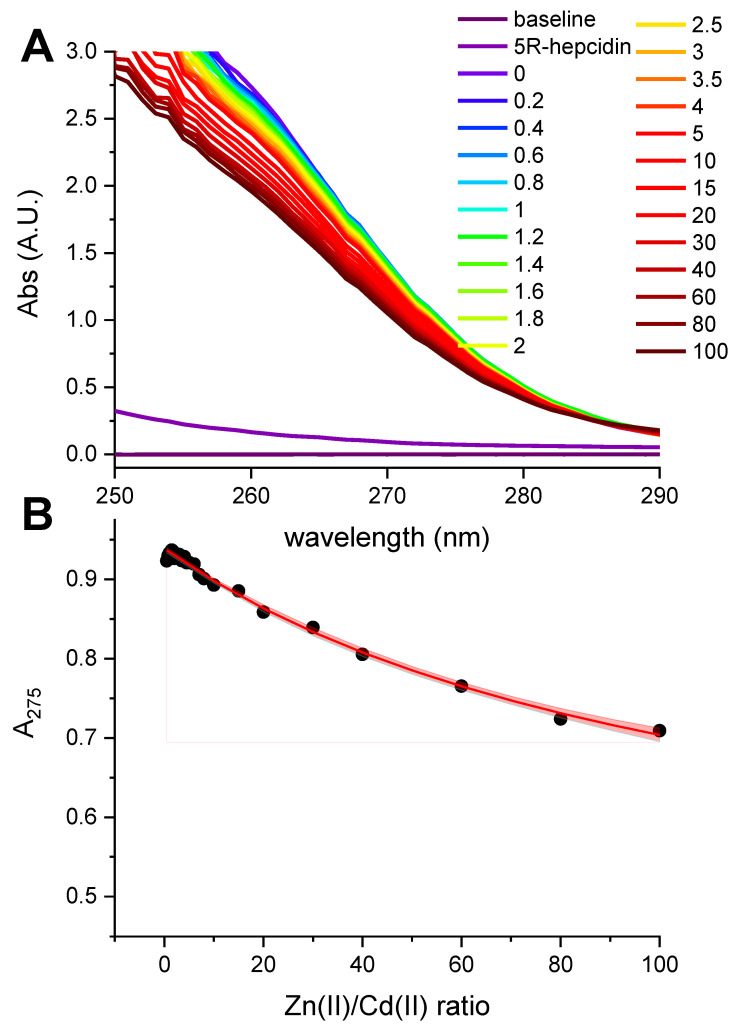
(**A**). 100 μM Cd_2_5R-hepcidin titrated with Zn(NO_3_)_2_ in 100 mM HEPES pH 7.4. Numbers in the legend denote molar excess of Zn(II) ions. (**B**). Titration curve at 275 nm and the fit for the competition reaction Cd_2_5R-hepcidin + Zn(II) = CdZn5R-hepcidin. The 95% confidence bands are marked in red.

Taking advantage of a much better solution stability of 5R-hepcidin, we then attempted to establish its relative affinity to Zn(II) and Cd(II) by titrating Cd_2_5R-hepcidin with Zn(II) ions, and vice versa. When Cd(II) ions were titrated to Zn_2_5R-hepcidin, the CT band rose as if there were no competing Zn(II) ions present (Figure 5). The apparent elevation of the band in the competition titration is due of the release of Zn(II) ions by Cd(II) competition (Figure 5B). In the reverse experiment, a high excess of Zn(II) ions was necessary to achieve partial displacement of one Cd(II) ion (Figure 6). The unitless competition constant calculated from this experiment for the reaction Cd_2_5R-hepcidin + Zn(II) = CdZn5R-hepcidin was 99 ± 14, meaning that ca. one hundredfold Zn(II) excess was necessary to displace one of the two Cd(II) ions bound to 5R-hepcidin. The displacement of only one Cd(II) ion was confirmed by the fact that the loss of the CT band asymptotically approached 50% of the initial value, corroborated by equal contribution of both Cd(II) ions to the CT band absorption (Figure 1). This value is lower than expected on the basis of affinity constant estimates presented above and may indicate the participation of His resides in the Zn(II) binding.

Next, we performed a test of the redox stability of the Cd(II):5R-hepcidin complex by exposing it to atmospheric oxygen. In theory, no disulfide bonds should be formed upon the Cd(II) protection, but the intrinsic dynamics of the coordination sites can lead to slow oxidation of the thiols. In the case of 5R-hepcidin, two Cd(II) equivalents prevented disulfide formation for more than 48 h (Figure S4). This experiment additionally confirmed the initial assumption that two Cd(II) ions were bound to all eight Cys residues of hepcidin.

**Figure 7:**
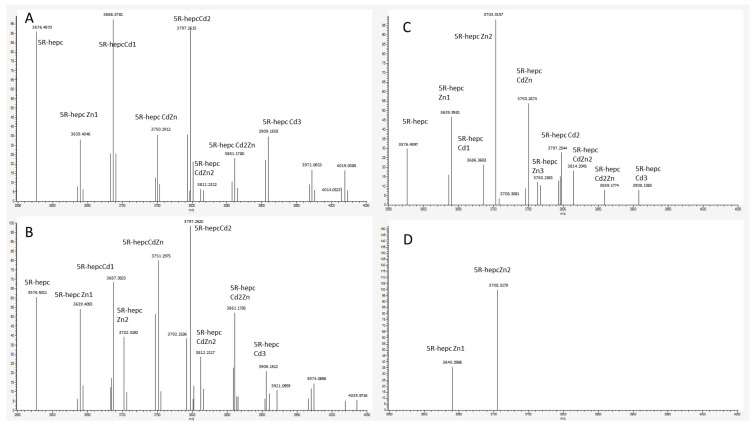
Deconvoluted Native MS spectra of 10 μM 5R-hepcidin and 20 μM Cd(II) acetate with the following concentrations of Zn_7_MT2A: (**A**) 1 μM, (**B**) 3 μM, (**C**) 10 μM, (**D**) 30 μM in ammonium acetate pH 7.4.

**Figure 8:**
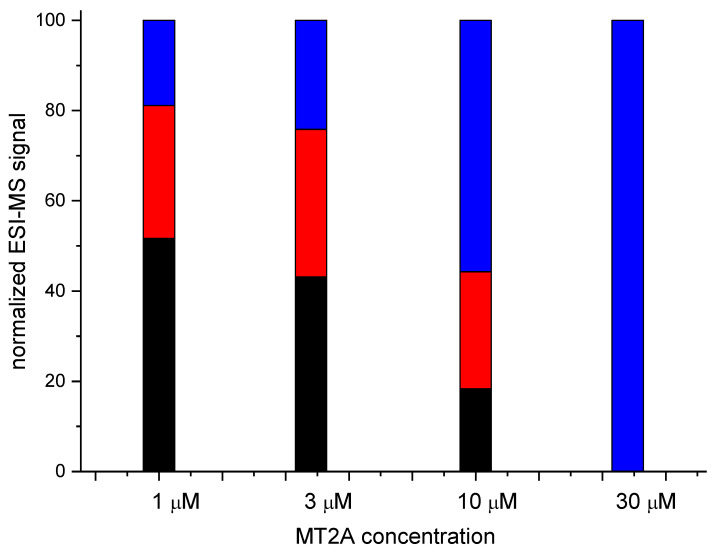
Quantitation obtained by careful integration of relevant signals from native MS experiment of 10 μM 5R-hepcidin and 20 μM Cd(II) acetate titrated with Zn_7_MT2A in ammonium acetate, pH 7.4. 5R-hepcidin (blue), Cd5R-hepcidin (red), Cd_2_5R-hepcidin (black).

In the final experiment native mass spectrometry was applied to a series of measurements with constant 5R-hepcidin and Cd(II) acetate and increasing amounts of Zn_7_MT2A in the presence of Zn(II) ion excess. MT2A is a natural Cd(II) ligand in cells exposed to cadmium. Despite the complicated character of this experiment which was aimed at reproducing physiological conditions to some extent, the Cd(II) complex of 5R-hepcidin could be monitored and quantified. The deconvoluted spectra are shown in Figure 7 (raw spectra in Figures S5 and S6) and the integrated signals of Cd_2_R5-hepcidin in Figure 8. Very clearly, 5R-hepcidin was able for compete for Cd(II) ions with MT2A.

## 3. Discussion

So far, the discussion of the direct role of hepcidin in metallobiochemistry has been limited to its mature, extracellular form in which thiol groups of all eight Cys residues are locked in specifically arranged disulfide bonds [9]. The ATCUN motif is the only metal-binding site in mature hepcidin-25 and its shorter model peptides, but its biological role remains to be seen [3,32]. In contrast, the set of experiments presented here demonstrates that the intracellular reduced form of hepcidin and/or its prepro- and pro-precursors is a strong ligand for biological Zn(II) and toxic Cd(II) ions. The combined study of spectroscopically and structurally equivalent hepcidin-25 and model 5R-hepcidin indicated that at physiological pH reduced hepcidin binds the Zn(II) ions with conditional log *K* of 10.1. This affinity is similar to the average affinity of Zn(II) ions to the proteome at large, 10.3 [33]. Considering that free Zn^2+^ ions do not exist intracellularly and instead the so-called “labile zinc” is in fact a heterogeneous pool of low-molecular-weight complexes, hypothesized to permeate the cell structures and serve as Zn(II) donors for proteins [34], hepcidin-25 may be at least partially metallated by physiological Zn(II) during its intracellular maturation. This notion has interesting implications for the mechanism of disulfide bond formation in the course of hepcidin maturation, and for the possible relationship between hepcidin/iron physiology and liver zinc status.

Furthermore, our studies indicated that reduced hepcidin is a potent Cd(II) ligand. The affinities of both of its sites for Cd(II) ions are similar to those of metallothionein [7]. The direct MS experiment demonstrated that hepcidin can actually compete with MT2 for Cd(II) ions. One should always be wary of mass spectrometry results as a substitute for solution studies, and take them with a grain of salt [31,35,36]. The abundance of Zn(II) ions residual from MT2A purification, which could not be fully removed from the MS system, could also distort the quantitative results, but the consistency of the competition experiment with in-solution titration experiments confirms that Cd(II) affinity of reduced hepcidin is high enough to compete with MT2.

The primary function of metallothionein is to control cytoplasmatic availability of Zn(II) and Cu(I) ions [7]. Detoxifying cadmium was likely not an evolutionary pressure before industrial times, which is true for all cadmium-related proteins in humans. Moreover, MT2A has multiple binding constants for Cd(II) [37], which means that although it is the reservoir for Cd(II) inside the cell, it is not an ultimate scavenger [27]. Moreover, our experiments confirm that Cd(II) can be retained by reduced hepcidin in the presence of MT2.

Despite some similarities of hepcidin and MTs regarding the Zn(II) and Cd(II) binding [37,38], they differ greatly. Biological experiments indicate that unlike the MTs, hepcidin apparently does not provide a cytoprotective effect. As opposed to MTs, hepcidin silencing decreases Cd(II) toxicity while overexpression of hepcidin decreases cell viability in the presence of cadmium [23]. Interaction of hepcidin and cadmium inside the cell (whether direct or indirect) results in the generation of reactive oxygen species. When it comes to the effect of hepcidin presence on iron-induced toxicity, the opposite was reported. Whether it is caused by hepcidin binding iron in vivo remains to be determined, as reduced hepcidin could apparently bind Fe^3+^ in the presence of TCEP [39]. The Cd(II)/Zn(II) competition experiment yielded an additional clue for the toxicity of Cd(II)-hepcidin. The pH titration indicated that the Zn(II) affinity to hepcidin-25 is ca. four orders of magnitude weaker than that of the weaker Cd(II) site (log K 10.1 vs. 14.1 at pH 7.4). In contrast, the titration of Zn(II) into Cd_2_5R-hepcidin demonstrated a ratio of only two orders of magnitude. One can speculate that the binding of the Cd(II) ion could pre-organize the peptide for stronger Zn(II) binding, on the metallothionein level (log K ~12). Thus, hepcidin could somehow contribute to cadmium toxicity by additionally interfering with zinc metabolism.

One could also hypothesize that cadmium can interfere with hepcidin maturation. Cadmium apparently does not enter the endoplasmic reticulum, where disulfides are normally formed, but it is present in the cytoplasm and nucleus [40]. It can also accumulate in the rough endoplasmatic reticulum membrane [41]. However, not all prohepcidin enters the secretory pathway, as it was found in the cytosol and nuclei of hepatic cells [14]. Conditions for that to occur have not yet been elucidated. Prohepcidin in blood correlates neither with iron loading, nor with inflammation, as normally hepcidin does [15,42]. It is correlated with renal failure [43], but has no clearly established function. Except for Cd-bound MTs, other sulfhydryl-rich proteins (which might include hepcidin) can transport cadmium from the liver to the kidneys [44]. We tested that Cd(II):hepcidin complex withstands atmospheric oxygen for at least 48 h (Figure S4). This covers the hepcidin half-life in blood of one day [45]. Our chemical proof-of-principle experiment thus demonstrated the resilience of the Cd(II) complex in oxidizing conditions. Therefore, it would be theoretically possible for improperly matured hepcidin to retain Cd(II) in blood.

We can safely conclude that at least partial exposure of hepcidin or prohepcidin to cadmium can lead to metalation of the peptide. Whether it results in improper folding and exporting of Cd-prohepcidin remains to be further investigated. One method that this could be explored is monitoring prohepcidin’s expression and secretion after cadmium exposure.

## 4. Materials and Methods

### 4.1. Materials

Dimethylformamid (DMF) was purchased from Carl Roth, Karlsruhe, Germany. Acetonitrile was obtained from Avantor POCH, Gliwice, PL. Peptide synthesis building blocks, Trifluoroacetic acid (TFA), Piperidine, HBTU, N,N-Diisopropylethylamine (DIEA), triisopropylsilane (TIS), and 1,2-Ethanedithiol (EDT) were from Merck, Darmstadt, Germany. 4-(2-Pyridylazo)resorcinol (PAR), HEPES buffer, 5,5′-Dithiobis(2-nitrobenzoic acid) (DTNB), Tris(2-carboxyethyl)phosphine hydrochloride (TCEP), and ammonium acetate were purchased from Sigma-Aldrich, St. Louis, MO, USA. Fmoc Thr(t-Bu) TentaGel S PHB resin was from Rapp polymere, Tübingen, Germany.

### 4.2. Methods

Peptide synthesis was carried out with a standard Fmoc-SPPS protocol using Liberty1 synthesizer (CEM) [46]. Peptides were purified with an acetonitrile:H_2_O gradient with 0.1% TFA and the purity was confirmed using ESI-QToF MS on Premier (Waters, Milford, MA, USA).

Expression and purification of metallothionein MT2A from Addgene plasmid ID 105,693 was carried out as before [47]. Concentration of metals was determined using PAR [48] and peptides using DTNB assay [49]. Metal titrations were carried out in duplicate on a Cary 50 Bio spectrophotometer (Varian) and a J-815 spectropolarimeter (Jasco). Whenever no TCEP was used, all titrations were performed in an anaerobic chamber flushed constantly with N_2_. The titrations presented are those with the lower peptide oxidation, according to the DTNB assay. In the air oxidation experiment, reagents were mixed in an anaerobic chamber, pH was adjusted with ammonia and/or formic acid, and the reagents were then taken to normal atmosphere and gently mixed. Titrations were analyzed using Origin2019. The p*K* values and standard deviations were determined by the embedded non-linear fitting algorithm. ESI-MS spectra were recorded on Premier ESI-QToF MS (Waters). All measurements were performed in the positive-ion mode. A source temperature of 80 °C was used for a complete desolvation of the peptide ions. The cone voltage was 30 V. The transmission of the ions was optimized on the quadrupole for the mass range 300 to 2000 *m*/*z*. Mass spectra were accumulated over 2 min to improve the signal-to-noise ratio. The sample flow was 20 μL/min.

Native MS was performed on a Q-Exactive UHMR Hybrid Quadrupole-Orbitrap mass spectrometer (Thermo Fisher Scientific, Waltham, MA, USA). Target peptides were prepared by dissolving peptides in 20 mM ammonium acetate pH 7.4 in 10 μM concentration. To each sample, cadmium acetate was added to reach a 2:1 ratio, followed by a differing concentration of the competing ligand. Measurements started after a 1 h delay to allow for reaching equilibrium. Samples were introduced into the mass spectrometer with a syringe pump using a 10 μL/min flow rate, by electrospray ionization using the positive mode in the HESI source. MS measurements were conducted under the following settings: desolvation voltage −20 V, capillary temperature 320 °C, detector m/z optimization low m/z, ion transfer optimization to low m/z. The RF applied throughout the instrument were set to 150 Vp-p for injection flatapole, 300 Vp-p for bent flatapole, 250 for transfer multipole and HCD cell, and 2300 for C-trap. The ion transfer optics were 5 V for injection flatapole, 4 V for intel flatapole, 2 V for bent flatapole, and 0 V for transfer multipole. Integration of the resulting peaks was achieved with the built-in software FreeStyle 1.4 (Thermo Scientific, Waltham, MA, USA).

## Data Availability

The data presented in this study are available on request from the corresponding author. The data are stored offline due to online security reasons.

## Supplementary Materials

The following supporting information can be downloaded at: https://www.mdpi.com/article/10.3390/ijms232415483/s1.
