# Supplementary material for: An Overlooked Hepcidin–Cadmium Connection
Source: Int J Mol Sci. 2022 Dec 7;23(24):15483. doi: 10.3390/ijms232415483 (PMC9779829; doi:10.3390/ijms232415483)
Supplement: Supplementary file 1 [file ijms-23-15483-s001.zip › ijms-1997898-supplementary.pdf]

SUPPORTING INFORMATION

An Overlooked Hepcidin-Cadmium Connection

Dawid Płonka [1], Marta D. Wiśniewska [1], Manuel D. Peris-Díaz [2], Artur Krężel [2], Arkadiusz M. Bonna [1] and Wojciech Bal [1,*]

[1] Institute of Biochemistry and Biophysics, Polish Academy of Sciences, Pawińskiego 5a, 02-106 Warsaw, Poland

[2] Department of Chemical Biology, Faculty of Biotechnology, University of Wrocław, Joliot-Curie 14a, 50-383 Wrocław, Poland

* Correspondence: wbal@ibb.waw.pl

Supporting figures

**MT2**	KS**CC**S**CC**PVG**C**AK**C**AQG**C**I**C**KGASDK**C**S**CC**A
**hepcidin-25**	DTHFPI**C**IF**CC**G**CC**HRSK**C**GM**CC**KT
**5R-hepcidin**	RRRRRDTHFPI**C**IF**CC**G**CC**HRSK**C**GM**CC**KT

Scheme S1. Comparison of sequences of α-domain of human metallothionein 2, hepcidin-25 and 5R-hepcidin (Cys residues set in bold).

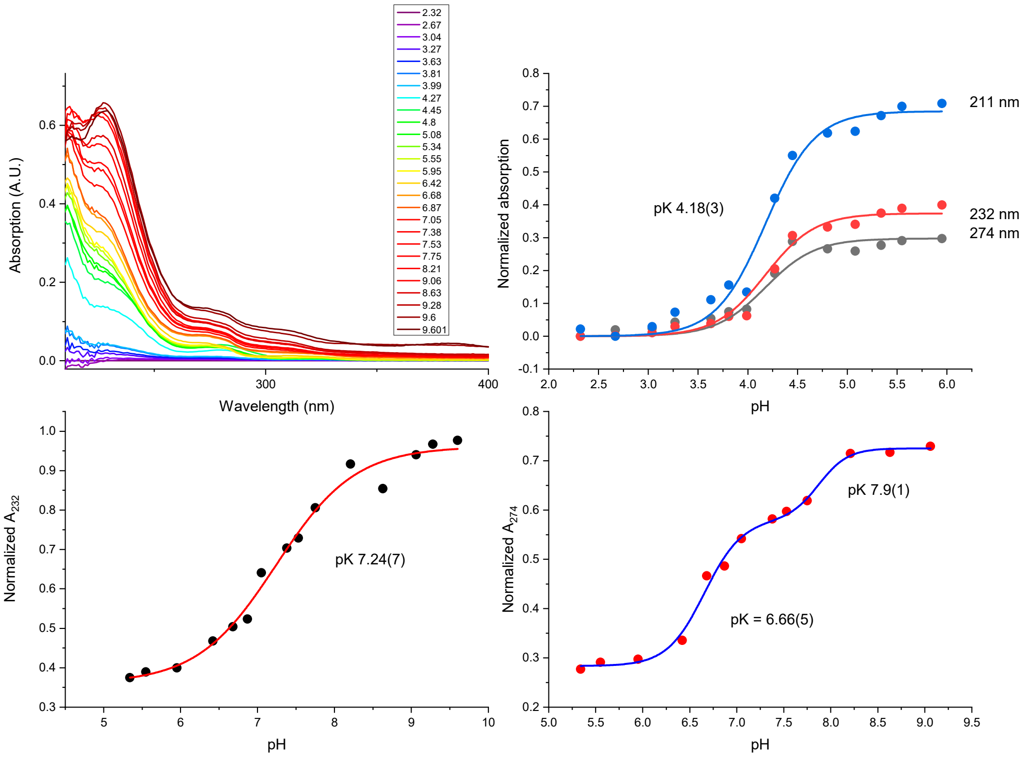

Figure S1. The UV pH-metric titration of 20 μM reduced 5R-hepcidin and the apparent p*K* values derived at various characteristic wavelengths.

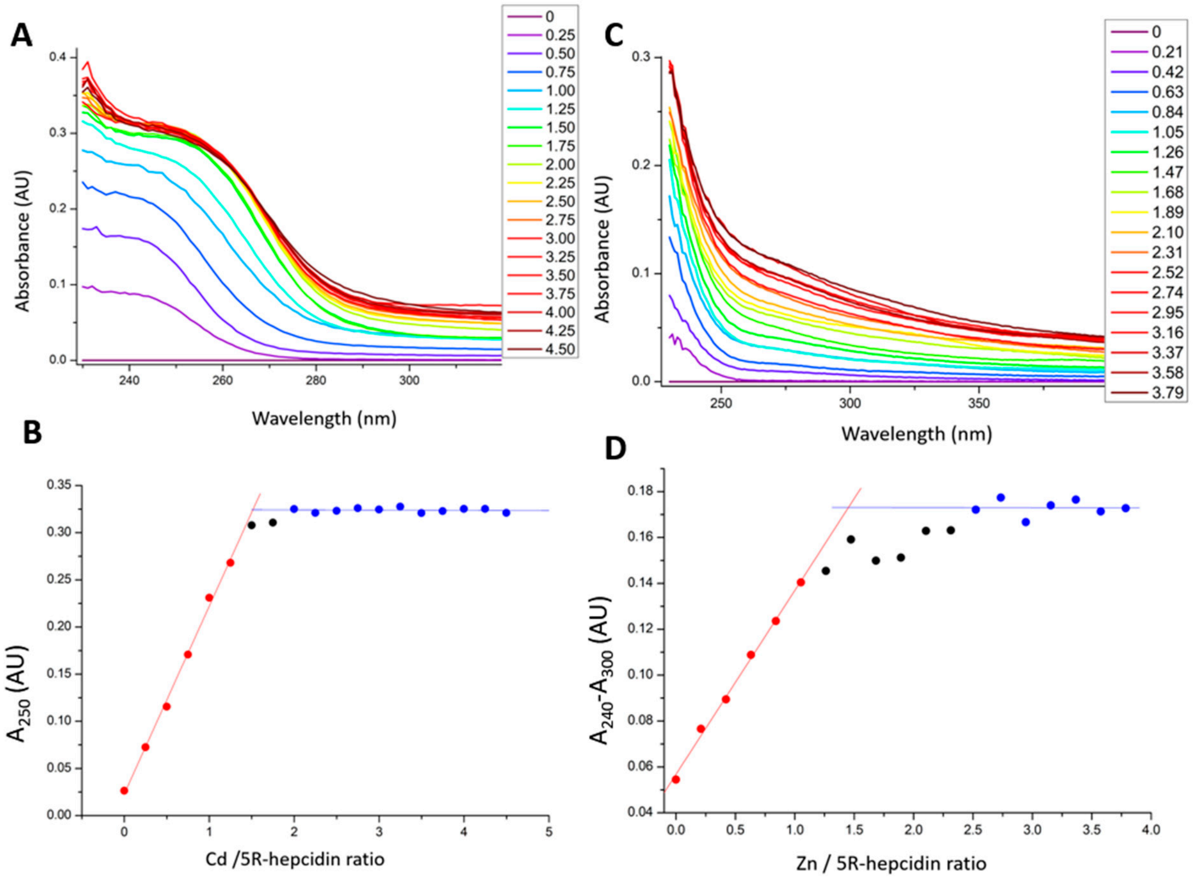

Figure S2. 10 µM 5R-hepcidin in 50 mM HEPES pH 7.4, with *I*=100mM established by NaCl, titrated with cadmium acetate, (A) presents the spectra, (B) presents the titration curve at 250 nm; and $ZnCl_2$, (C) presents the spectra, (D) presents the differential titration curve at 240 nm – 300 nm. Cd(II) and Zn(II) to peptide ratios are color coded on the plots.

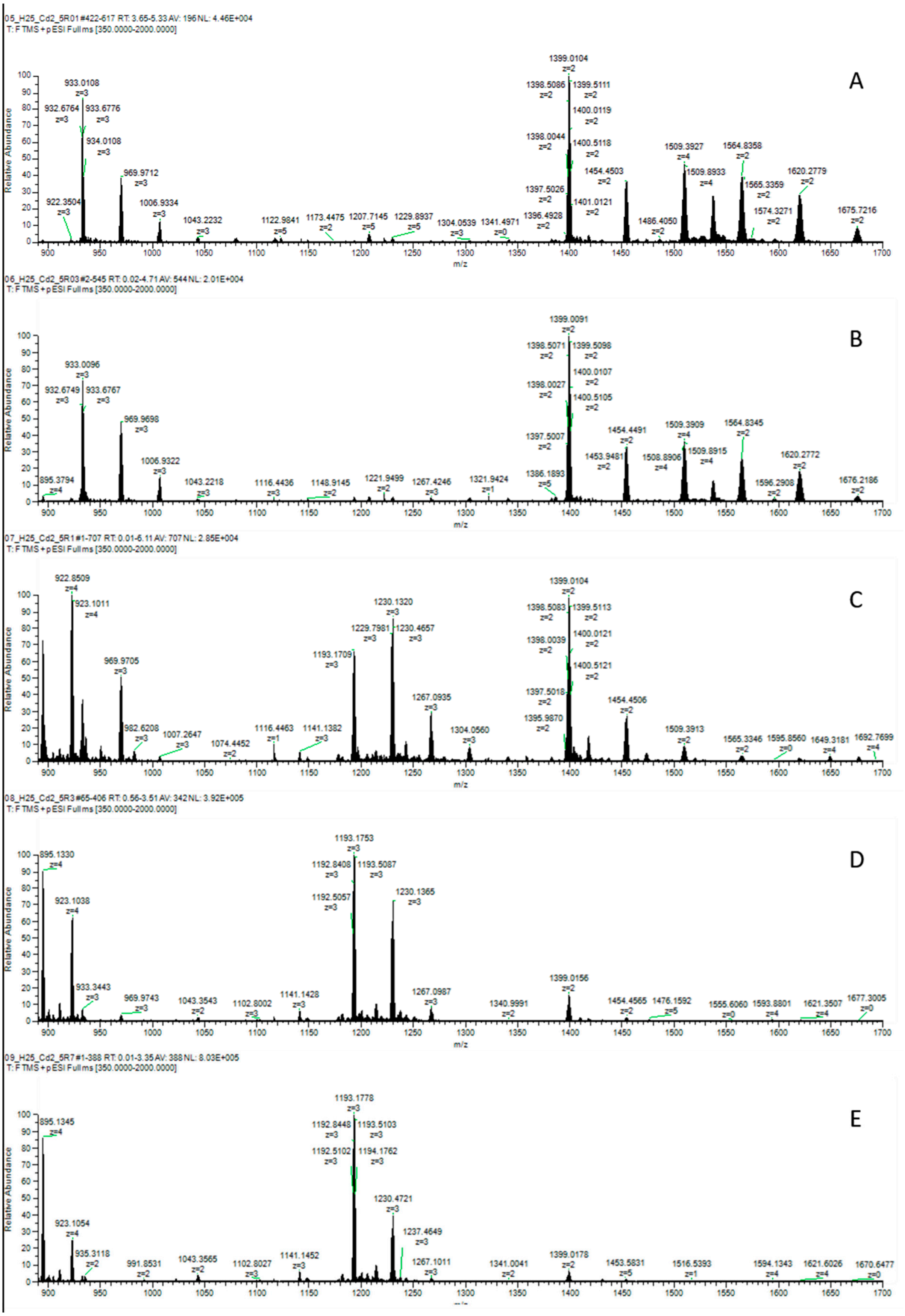

Figure S3. Native mass spectra of 10 μM Hepcidin-25 with 20 μM Cd(II) acetate with varying concentration of 5R-hepcidin: A) 1 μM, B) 3 μM, C) 10 μM, D) 30 μM, E) 70 μM in ammonium acetate pH 7.4.

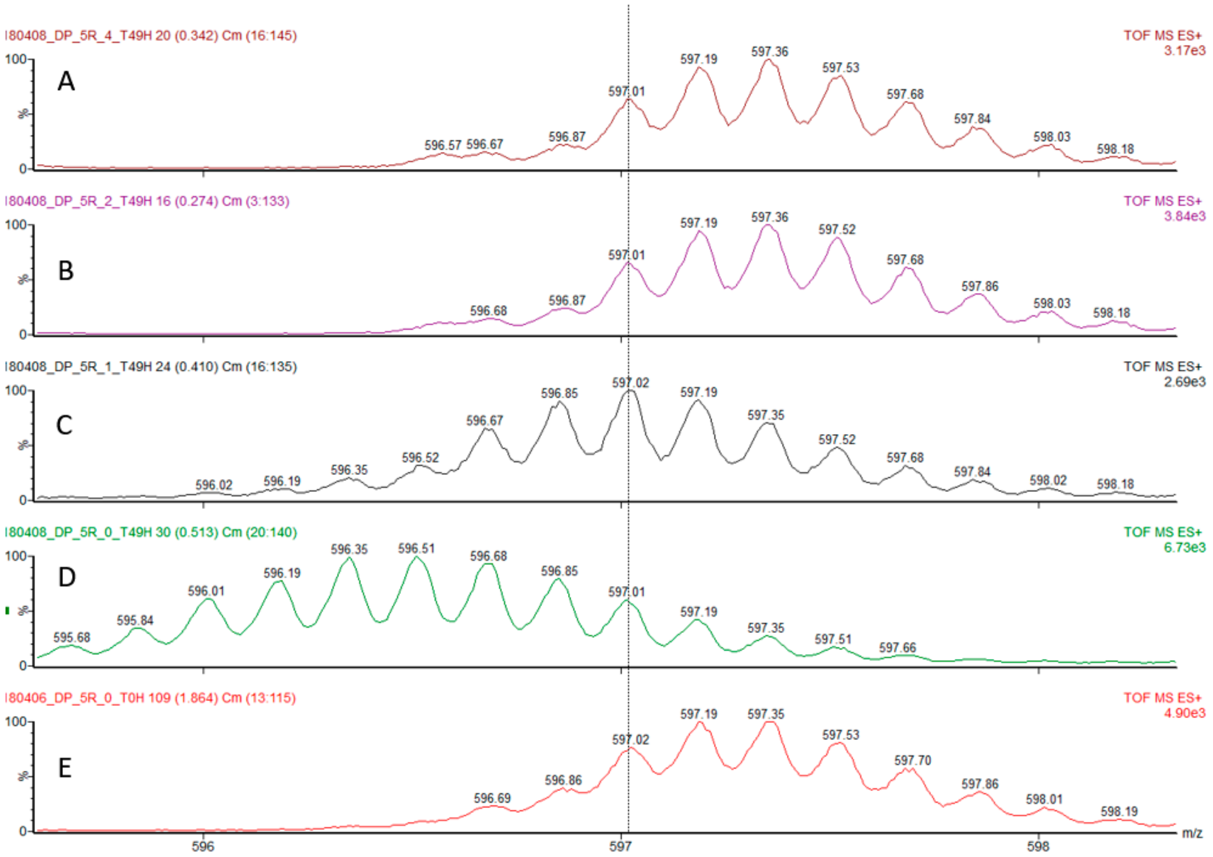

Figure S4. 5R-hepcidin (100 μM) incubated with 4, 2, 1 and 0 Cd(II) equivalents (A, B, C and D respectively) for 49 hours in pH 6, with the initial sample presented in E. The disulfide formation was monitored at ESI-QToF MS at 6+ charged peptide ions $[M+6H]^{6+}$. The isotopic distribution shift to left, indicating the loss of two H atoms for each disulfide formed. The monoisotopic peak of reduced peptide is marked with the dotted line. Small peaks left from the dotted line (one S-S bond) in all samples occurred during sample preparation process.

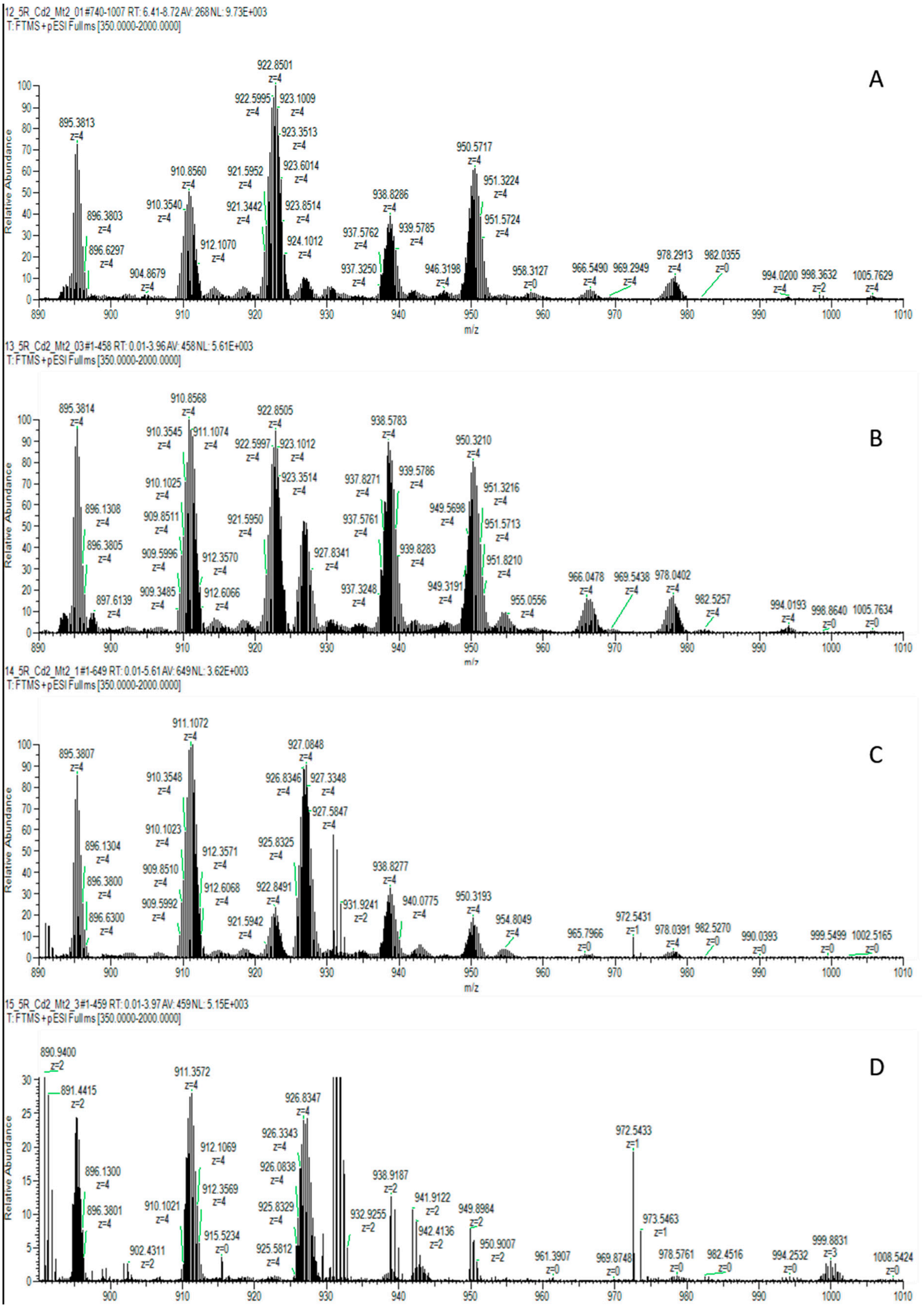

Figure S5. Native mass spectra of 10 µM 5R-hepcidin with 20 µM Cd(II) acetate with varying concentration of metallothionein MT2A: A) 1 µM, B) 3 µM, C) 10 µM, D) 30 µM in ammonium acetate pH 7.4 Due to high noise and differing intensities, for better clarity only fragment for 5R-hepcidin is shown, the fragment with peaks associated with MT2A is shown in Fig. S6.

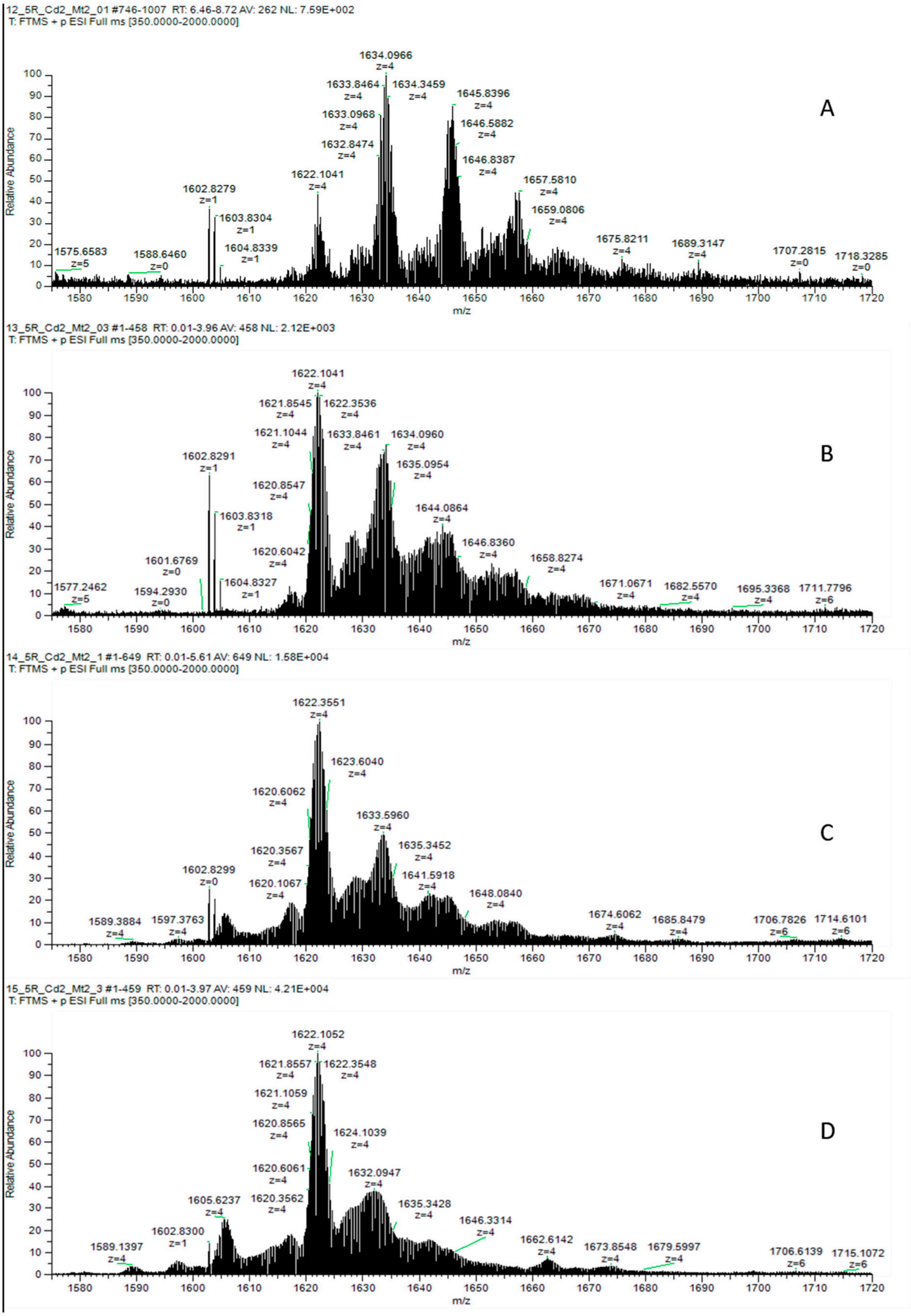

Figure S6. Native mass spectra of 10 µM 5R-hepcidin with 20 µM Cd(II) acetate with varying concentration of metallothionein MT2A: A) 1 µM, B) 3 µM, C) 10 µM, D) 30 µM in ammonium acetate pH 7.4 Due to high noise and differing intensities, for better clarity only peaks associated with MT2A is shown, the spectrum fragment with peaks associated with 5R-hepcidin is shown in Fig. S5.
